# An outbreak of *Providencia rettgeri* bacteremia at a *Ptyas mucosus* farm in Hainan, China

**DOI:** 10.3389/fmicb.2024.1353603

**Published:** 2024-07-11

**Authors:** Lixia Fan, Jiwen Pan, Jifeng Zeng, Guiying Guo, Nou Yang, Xuesong Li, Muhammad Nafees Ur Rehman, Jiping Zheng

**Affiliations:** ^1^Lab of Microbial Engineering (Infection and Immunity), School of Life and Health Sciences, Hainan University, Haikou, China; ^2^Institute of Tropical Bioscience and Biotechnology, China Academy of Tropical Agricultural Sciences, Haikou, Hainan, China; ^3^Hainan Province Key Laboratory of One Health, Collaborative Innovation Center of One Health, Hainan University, Haikou, China; ^4^School of Chemistry and Chemical Engineering, Hainan University, Haikou, China

**Keywords:** *Ptyas mucosus*, *Providencia rettgeri*, histopathology, virulence, resistance

## Abstract

**Aim:**

To describe the histopathology and etiology of an outbreak of respiratory disease at a *Ptyas mucosus* farm in Hainan, China.

**Methods and results:**

The etiology was confirmed by gross examination and microscopic analysis. The bacterial isolates from blood and internal organs were identified by biochemical analysis and 16S rRNA gene sequencing. The virulence and antibiotic resistance characteristics of the isolates were further demonstrated by polymerase chain reaction (PCR), disk diffusion testing, and LD_50_ analysis in Kunming mice. Histopathological analysis of the diseased *P. mucosus* revealed systemic lesions, including severe airway obstruction with large numbers of inflammatory cells and cellulose exudates in the lungs; severe multifocal hepatocyte vacuolar degeneration and necrosis in the liver with excessive inflammatory exudates and chronic granuloma; splenic hemorrhage and partial loss of splenic structure; and renal vascular and interstitial congestion. *Providencia rettgeri* was isolated from the blood and multiple internal organs (liver, spleen, kidneys, and lungs). All examined isolates (H1, H4, and H13) were multidrug-resistant but sensitive to four antibiotics—cefepime, imipenem, chloramphenicol, and ciprofloxacin. Both H1 and H4 carried five resistance genes [*bla*_OXA_, *tet*(A), *tet*(B), *tet*(E), and *aac* (3)-IIa], whereas H13 only carried the *tet*(A) gene. The dominant virulence pattern of the three isolates was *hly*A + *Zap*A + *lux*S + *rsb*A. The virulence of H1 strain was tested, and its 50% lethal dose (LD_50_) in mice was 2.29 × 10^8^ CFU ml^−1^.

**Conclusion:**

To our knowledge, this is the first study to describe an outbreak of bacteremia caused by *P. rettgeri* in farmed rat snakes.

**Significance and impact of the study:**

The results highlight that *P. rettgeri* is an emerging bacterial pathogen in farmed reptiles.

## 1 Introduction

*Ptyas mucosus*, also known as oriental rat snake or mucosal rat snake, is a common species of colubrid snakes used in the international skin trade (Zhou et al., 2016). Breeding has been an effective method for conservation and commercial skin trade in many Asian countries including China (Aust et al., 2017), since the reptile was listed in Appendix II of the Convention on International Trade in Endangered Species of Wild Fauna and Flora in January 1990. Death due to infectious diseases often occurs, especially respiratory diseases, and this remains the main threat in the snake farming industry (Su et al., 2020; Comolli and Divers, 2021). The causes of snake pneumonia include viral, bacterial, fungal, and parasite infections, among which bacterial pneumonia in snakes is more common (Xia et al., 2022). *Providencia rettgeri* is an opportunistic pathogen that has been isolated from some reptiles such as crocodile monitor Var*anus salvadorii* (Kycko et al., 2013), soft-shelled turtle *Trionyx sinensis* (Fan et al., 2021), red-eared slider turtle *Trachemys scripta*, and turtle *Trachemys scripta* (Ye et al., 2020). However, few studies have examined this issue in *P. mucosus* (Purwaningsih, 2011; Wang et al., 2011; Bino Sundar et al., 2015).

In October 2022, a respiratory disease outbreak occurred on a mucosal rat snake farm in Qionghai, Hainan, China. The main signs were mouthfuls of mucus, nasal discharge, open mouth breathing with “gurgling” sounds, and wheezing, and most of the snakes displayed lethargy and a lack of appetite. In this study, we describe the histopathology and reveal the etiology of the respiratory disease in mucosal rat snakes to help farmers effectively treat and prevent such diseases in the snake industry.

## 2 Materials and methods

### 2.1 Necropsy and histopathology

Three lethargic mucosal rate snakes were sent to the Lab of Microbial Engineering (Infection and Immunity), School of Life and Health Sciences, Hainan University. Gross post-mortem and histopathological examination were performed as previously described (Rehman et al., 2021). In brief, the snakes were necropsied for primary visual examination, and the internal organs including the lungs, liver, spleen, kidneys, heart, and stomach were harvested for paraffin sectioning. Finally, the histological features were observed at 100× and 400× under a light microscope.

### 2.2 Bacterial examination

Bacterial isolation as previously described by our group (Rehman et al., 2021). The tissue samples were collected by inserting a sterile loop into the aforementioned internal organs. We cultured bacterial colonies by streaking the samples onto plates containing brain-heart infusion (BHI) medium and incubating them at 37°C for 18 h. To identify the isolates, their morphological features were observed by microscopy at 400× and 1,000× following Gram staining, and their physical and biochemical characteristics were analyzed using an API20E automatic bacterial identification system (bioMėrieux, Marcy l’Etoile, France). Phylogenetic analysis was performed using MEGA7.0 software based on 16S rRNA gene amplification, sequencing, and GenBank Basic Local Alignment Search Tool analysis, as described in our previous studies (Zheng et al., 2018; Rehman et al., 2021).

### 2.3 Antimicrobial susceptibility assay

The Kirby–Bauer method (Jacobs et al., 1979) was used to conduct antibiotic susceptibility testing for 12 common antimicrobials (Oxoid, UK), and the results were statistically analyzed in accordance with the evaluation criteria for antimicrobial susceptibility formulated by the Clinical and Laboratory Standards Institute (CLSI) (James et al., 2023).

### 2.4 Genotype assay for resistance and virulence

The resistance and virulence genes were screened using polymerase chain reaction (PCR) amplification, as previously reported by our group (Rehman et al., 2021). The primers and amplicon sizes for 12 antibiotic-resistance genes are shown in Supplementary Table S1. These genes code for β-lactamase resistance (*bla*_TEM_, *bla*_SHV_, *bla*_CTX-M_, and *bla*_OXA_); tetracycline resistance (*tet*(A), *tet*(B), and *tet*(E)); and aminoglycoside resistance (*aph*AI-IAB, *aac* (3)-, *aac* (6′)-lb, *Str*A-B, and *arm*A) (Pathirana et al., 2018). Supplementary Table S2 represents the primers and their amplicon sizes for seven virulence genes (*ure*C, *rsb*A, *mrp*A, *hmp*A, *hly*A, *zap*A, and *lux*S) (Abbas et al., 2015; Pathirana et al., 2018). PCR products were detected using 1% agarose gel electrophoresis. Genotypic patterns were determined according to the detected gene compositions.

### 2.5 Mouse experiments

Cells were harvested at the exponential growth stage and then re-suspended in phosphate-buffered saline (PBS) to an optical density of 1.0 at 600 nm. Viable cells were counted as colony-forming units (CFU). Thirty specific pathogen-free 7-week-old Kunming mice (KM) (female:male = 1:1; Hunan STA Laboratory Animal Co. Ltd., Changsha, China) were randomly divided into six groups (*n* = 5 per group). All five groups were injected intraperitoneally with 30 μL cells (1 × 10^5^ to 1 × 10^9^ CFU) in 1:10 dilutions, while the control group mice were inoculated with PBS. Mortality was recorded daily after administration. The half-maximal lethal dose (LD_50_) was measured using probit analysis (Finney, 1985) on SPSS 24.0 software (IBM Corp., Armonk, NY, USA). To confirm the experimental infection, heart blood (blood of the heart), liver, and lungs were collected immediately from the dead mice for bacterial isolation. The Ethics Committee for Animal Laboratories, Hainan University approved all protocols.

## 3 Results

### 3.1 Necropsy and histopathology

**Figure 1:**
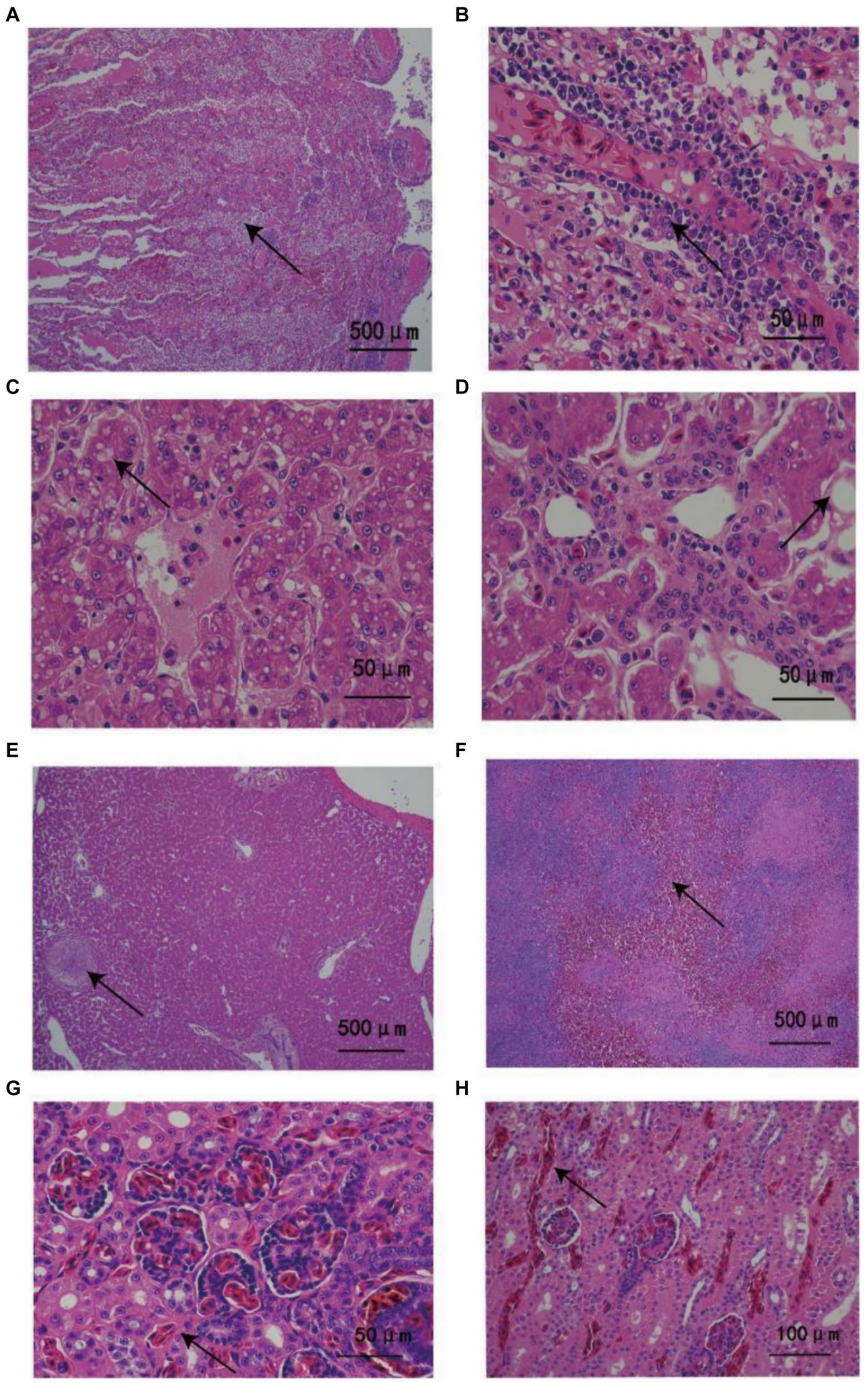
Histopathological changes in *Ptyas mucosus* infected with *Providencia rettgeri.* **(A)** Most of the airway was blocked by large numbers of inflammatory cells and fibrous exudates (arrows). **(B)** Large numbers of inflammatory cells invaded the lung tissues (arrow). **(C,D)** The liver had diffused vacuolar degeneration as well as a large amount of surrounding inflammatory exudates (arrow). **(E)** Chronic granuloma was detected in the parenchyma of the liver with caseous nodules (arrow) accompanied by thickening of the surrounding blood vessel wall. **(F)** Spleen bleeding, partial spleen structure loss (arrow). **(G,H)** Renal vascular and interstitial congestion with inflammatory cell infiltration (arrow).

The weight and length of the three snakes was 1.24, 1.48, and 1.51 kg and 1.53, 1.6, and 1.68 m, respectively. Obvious abnormal fluid was observed in the mouth and choanal slit, but there was no evidence of scarring, irregular pigmentation, wound, or ectoparasites. Histopathological analysis revealed similar lesions among the dead snakes. Large numbers of inflammatory cells and cellulose exudates were detected in the lungs (Figures 1A,B). The livers of the animals exhibited severe hepatocyte vacuolar degeneration, multifocal necrosis, and multiple granulomas (Figures 1C–E). Splenic hemorrhage accompanied by partial structural loss was also observed (Figure 1F). The kidneys showed severe hyperemia and slight vesicular degeneration of the renal tubular epithelial cells (Figures 1G,H).

### 3.2 Bacteria examination

Similar colonies were grown from the lung, trachea, blood, liver, kidney, and spleen samples on BHI plates. Three strains named H13 (GenBank accession number: OQ359956), H4 (GenBank accession number: OQ359955), and H1 (GenBank accession number: OQ359954) were isolated from the lung, trachea, and blood of different snakes (Table 1). All the isolates were gram-negative and rod-shaped. With regard to the biochemical traits, they exhibited the ability to produce urease; utilize sodium citrate; yield positive results for indole production; and metabolize tryptophan, mannitol, glucose, and inositol (Supplementary Table S3). Therefore, we preliminarily identified them as *P. rettgeri*.

**Table 1:** Resistance patterns of the three *Providencia rettgeri* isolates.

Antibiotic		MIC (μg ml^−1^)		
Group	Drug	H1 (blood)	H4 (trachea)	H13 (lung)
Chloramphenicol	Chloramphenicol	≤8 (S)	≤8 (S)	≤8 (S)
Carbapenems	Imipenem	≤1 (S)	≤1 (S)	≤1 (S)
Cephalosporin	Cefepime	≤2 (S)	≤2 (S)	≤2 (S)
	Cefazolin	≥32 (R)	≤16 (S)	≤16 (S)
Fluoroquinolones	Ciprofloxacin	≤0.06 (S)	≤0.06(S)	≤0.06 (S)
	Ofloxacin	≥16 (R)	4 (I)	4 (I)
Penicillin	Penicillin	≥32 (R)	16 (I)	≥32 (R)
Aminoglycosides	Gentamicin	≥2 (R)	≤0.5 (S)	≥2 (R)
Macrolides	Azithromycin	≥32 (R)	≥32 (R)	16 (I)
Tetracycline	Tetracycline	≥16 (R)	≥16 (R)	≥16 (R)
Cationic polypeptide	Polymyxin B	≥4 (R)	≥4 (R)	≥4 (R)
Sulfonamide	Sulphamethoxazole	≥128 (R)	≥128 (R)	≥128 (R)

R, Drug resistance; I, Intermediate; S, Sensitivity. Breakpoints based on CLSI M100 performance standards for antimicrobial susceptibility testing.

**Figure 2:**
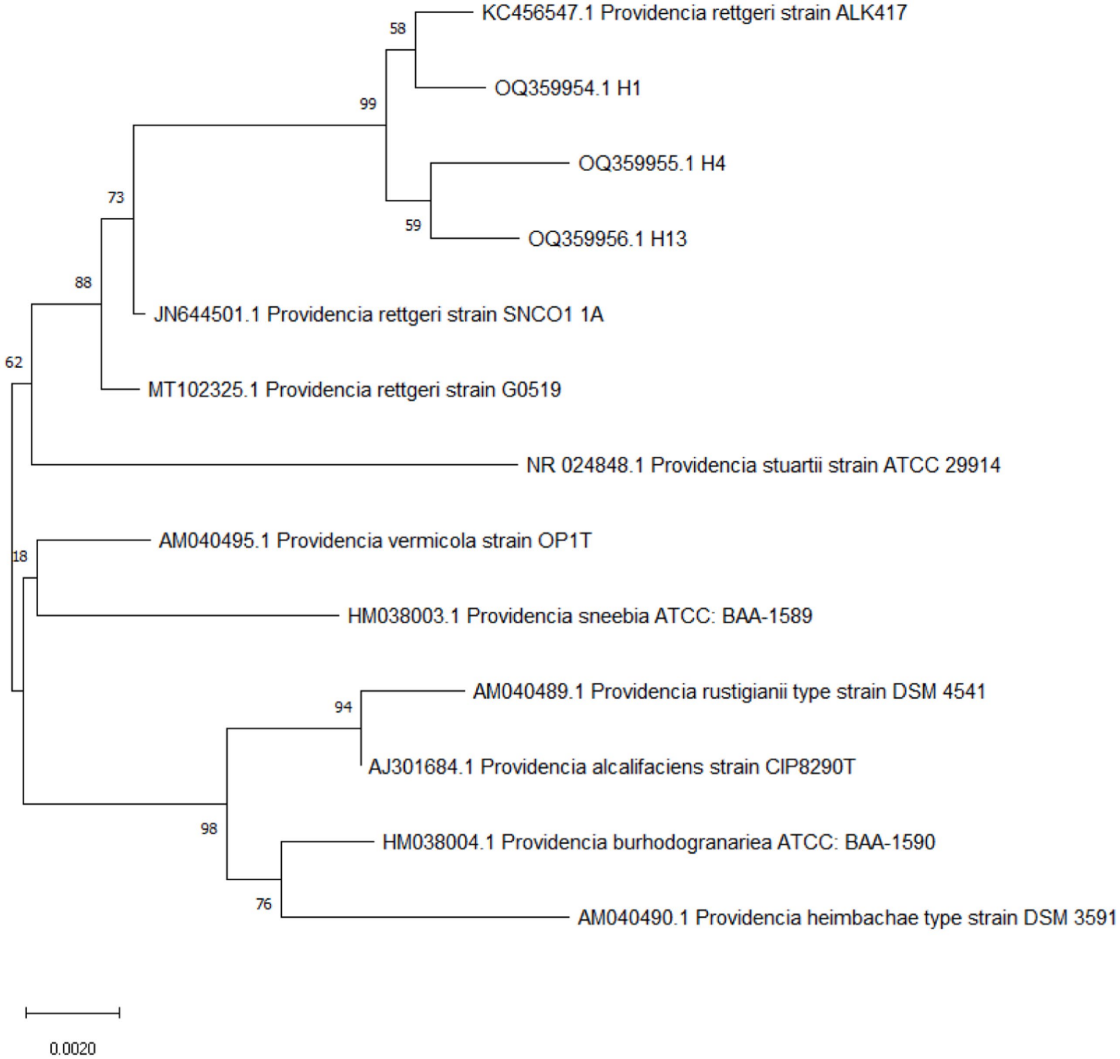
16S rRNA phylogenetic tree of the isolated strains.

The three strain results of 16S rRNA gene sequencing were searched by BLAST on the NCBI website. The comparison results showed that H1 and ALK417 were clustered into one strand, and H4 and H13 were clustered into another strand. Then, the two strands clustered with *P. rettgeri* G0519 coming from reptiles into one strand (Figure 2). Based on the biochemical traits, bacterial 16S rRNA gene sequence analysis, and phylogenetic tree construction, bacteria H1, H4, and H13 were identified as *P. rettgeri* (Figure 2).

### 3.3 Antimicrobial susceptibility tests

The three isolates H1, H4, and H13 were tested against 12 agents from 11 antibiotic classes (Table 1), in which seven antibiotics were resistant to polymyxin B, sulfamethoxazole, and tetracycline, indicating multi-drug-resistance (MDR). However, only four drugs were found to be susceptible to the isolates, namely imipenem, chloramphenicol, cefepime, and ciprofloxacin. Compared with the susceptibilities of the three isolates, H1 was the most drug-resistant isolate, exhibiting high resistance to penicillin and cefazolin, whereas H4 was the most drug-sensitive strain, displaying high sensitivity to penicillin and cefazolin.

### 3.4 Genotype assay for resistance and virulence

According to PCR analysis of the 12 resistance genes from three groups (Table 2) and seven virulence genes (Table 3), H1 and H4 shared the same resistance genotype comprising five genes, namely the oxacillinase (OXA, class D β-lactamase)-resistance gene *bla*_OXA_; tetracycline-resistance genes *tet*(A), *tet*(B), and *tet*(E); and aminoglycoside N (3)-acetyltransferase-resistance gene *aac* (3)-IIa. Conversely, only a single gene (*tet*(A)) was detected in H13. Meanwhile, all three isolates had the same virulence pattern of *hly*A + *Zap*A + *lux*S + *rsb*A, and they lacked three invasive genes, namely the major structural subunit of the mannose-resistant *Proteus*-like (MR/P) fimbriae gene *mrp*A, the urease alpha subunit gene *ure*C, and the calcium-independent hemolysin gene *hpm*A.

**Table 2:** Distribution of resistance genes in the three *Providencia rettgeri* isolates.

Resistance gene	β-lactamase				Tetracycline			Aminoglycoside				
	*bla*_TEM_	*bla*_SHV_	*bla*_CTX-M_	*bla*_OXA_	Tet(A)	Tet(B)	Tet(E)	*aph*AI-IAB	*aac* (3)- IIa	*aac* (6′)-lb	*str*A-B	armA
Isolate (100%) H1	−	−	−	+	+	+	+	−	+	−	−	−
Isolate (100%) H4	−	−	−	+	+	+	+	−	+	−	−	−
Isolate (100% H13	−	−	−	−	+	−	−	−	−	−	−	−

**Table 3:** Distribution of virulence genes in the three *Providencia rettgeri* isolates.

Virulence gene	Description (reference)	Isolate (100%)
*mrpA*	The main subunit of MR/P fimbriae, required to establish infection (Lane et al., 2009)	−
*ureC*	Urease alpha subunit, to hydrolyze urea into ammonia and carbon dioxide, required for bladder stone formation (Schaffer et al., 2016)	−
*hpmA*	Calcium-independent hemolysin, cytolytic activity and cytotoxicity (Alamuri et al., 2009)	−
*hlyA*	Calcium-dependent hemolysin, cytolytic activity and cytotoxicity (Welch, 1987)	+
*zap*A	Metalloprotease, required to degrade a number of substrates, including immunoglobulin A (IgA) and defensins of the host’s immune system, and promote bacterial surface migration and colonization (Walker et al., 1999; Phan et al., 2008)	+
*rsbA*	A repressor of swarming and virulence factor expression, modulation of swarming and virulence (Liaw et al., 2004)	+
*luxS*	S-ribosylhomocysteine lyase, formation of biofilm and expression of virulence factors (Schneider et al., 2002)	+

### 3.5 Mouse experiments

Based on its greater antimicrobial resistance but similar virulence as the other two isolates, H1 was chosen for further virulence analysis in a KM mouse model. The typical symptoms included extant hunched posture, lethargy, and a loss of appetite on day 2; death occurred in groups 1 and 2 on day 4 after infection (Table 4). In addition, H1 was re-isolated from the heart blood, livers, and lungs of dead mice via bacterial isolation and API20E analysis. The LD_50_ was calculated to be 2.29 × 10^8^ CFU ml^−1^ based on the 7-day mortality rate of the five groups, which indicated that H1 was pathogenic to the experimental KM mice.

**Table 4:** Mortality of Kunming mice intraperitoneally infected with *Providencia rettgeri* H1.

Group (*n* = 5 per group)	Dose 30 μL (CFU ml^−1^)	Death number (7 days)	Mortality (%)	LD_50_
1	2.76 × 10^9^	5	100	2.29 × 10^8^
2	2.76 × 10^8^	3	60	
3	2.76 × 10^7^	0	0	
4	2.76 × 10^6^	0	0	
5	2.76 × 10^5^	0	0	
CK	PBS	0	0	

## 4 Discussion

Although parasitic and fungal diseases are known to occur in *P. mucosus,* in this study, we primarily described an outbreak of bacteremia by *P. rettgeri* at a rat snake farm in Hainan, China (Lorch et al., 2016). As an opportunistic bacterial pathogen, *P. rettgeri* has been isolated from many domestic animals and humans, and causes respiratory diseases (Sharma et al., 2017), diarrhea, urinary tract infection (Barrios et al., 2013; Sagar et al., 2017), and encephalitis (Ladds et al., 1996). Furthermore, *P. rettgeri* can cause several diseases in Var*ancus salvadorii*, septicemia in *Trionyx sinensis* and *Trachemys scripta*, and likely even death in *Trachemys scripta* (Ye et al., 2020). The clinical characteristics of pneumonia and histopathological signs of extensive lesions in the lungs, liver, spleen, and kidneys in this study were consistent with the symptoms of fast respiration and lethargy, as well as the features of sepsis and shock on chest radiography in humans (Sharma et al., 2017).

In terms of the MDR, which is defined as resistance to three or more classes of antibiotics (Tada et al., 2014), all three isolates of *P. rettgeri* showed high-level MDR to four classes of antimicrobial agents, namely polypeptides, cationic polypeptides, sulphonamides, and tetracyclines. By comparing the tetracycline-resistance genes in isolates, H1 and H4 carried *tet*(A), *tet*(B), and *tet*(E) genes, whereas H3 only carried *tet*(A). This highlights the consequences of the historical misuse and overuse of antibiotics and warns that resistance is a very rampant occurrence and a serious concern across the entire ecosystem including wildlife, livestock, and humans. Fortunately, four drug types—cefepime (beta-lactam class, which interrupts bacterial cell-wall formation), imipenem (beta-lactam), chloramphenicol (a broad-spectrum antimicrobial agent that inhibits protein synthesis), and ciprofloxacin (fluoroquinolone class that inhibits DNA replication by inhibiting bacterial DNA topoisomerase and DNA-gyrase)—were practically useful and effective to prevent the outbreak of *P. rettgeri* infection at the snake farm in this study. Cephalosporins, ciprofloxacin, and lincomycin are often used in the snake industry, which is very consistent with the descriptions that the strains of *Providencia* spp. are susceptible to critically important antimicrobials of the fluoroquinolone class and beta-lactam class such as norfloxacin, imipenem, cefoxitin, and ceftazidime (Di et al., 2018).

Our findings showed that four of the seven tested virulence genes were recognized in the isolates (Table 3). The four genes were *hly*A, which encodes a calcium-dependent hemolysin, forming pores and channels between the lipid bilayer membranes and dissolves red blood cells and neutrophils (Welch, 1987); *zap*A, a gene that encodes a broad-spectrum metalloproteinase that can degrade a variety of immune factors and structural components of host cells (Walker et al., 1999; Phan et al., 2008); *rsb*A, which encodes a sensor protein that transmits environmental stimuli, and is also involved in stimulating the formation of biofilm and extracellular polysaccharides (Liaw et al., 2004); and *lux*S, a gene related to the automatic induction molecule AI-2 that mediates quorum sensing and biofilm formation (Schneider et al., 2002). Because evidence has shown that *hly*A and *zap*A are the most important genes involved in the immune evasion of many bacterial pathogens in urinary tract infections (Sagar et al., 2017), and that *luxS* and *rsbA* were the highest-frequency virulent genes of quorum sensing in *Proteus mirabilis* from urinary tract infection (Hussein et al., 2020), *P. rettgeri* has been recognized as an emerging nosocomial uropathogen and is notorious to treat due to escalating antibiotic resistance in clinical practice (Rajni et al., 2022). Furthermore, the high-level MDR resistance is also reflected in the isolates of *P. rettgeri* from farmed animals in this study. Therefore, selection of appropriate antibiotic therapy should be emphasized in both veterinary and medical settings to control the spread of infection in this species.

High-level MDR and the profile of virulence genes are two important factors in *Providencia* infection (Tada et al., 2014; Olaitan et al., 2016; Saavedra-Rojas et al., 2017; Di et al., 2018; Iwata et al., 2020; Piza-Buitrago et al., 2020). The comparison data of the LD_50_ value of 6.87 × 10^7^ CFU (30 μL, 2.29 × 10^8^ CFU ml^−1^) in this study with the LD_50_ value of 5.62 × 10^7^ cells in a previous study (Obayes and GAbd, 2013) have shown that *P. rettgeri* H1 is almost identical to the isolates from patients with urinary tract infections (UTIs), in terms of virulence by using a mouse model (Obayes and GAbd, 2013). These results suggest that *P. rettgeri* would be an environmental zoonotic pathogen with the ability to infect snakes, rats, and humans, because the isolates from *P. mucosus* in this study showed the closest relationship with indigenous strain ALK417 in soil (GenBank: KC456547.1) and strain SNCO1_1A from mosquito midgut microbial community (Schneider et al., 2013) (Figure 2).

In conclusion, this study primarily described the histopathology, biochemistry, virulence, and resistance of *P. rettgeri* in farmed *P. mucosus* with bacteremia, and highlighted that this species is a rare and neglected opportunistic wildlife pathogen. Given the complications of antibiotic therapy considering the high-level MDR of these isolates, one strategy would be to potentially fight and block the transmission of this species from the environment to animals and humans.

## Data availability statement

The datasets presented in this study can be found in online repositories. The names of the repository/repositories and accession number(s) can be found in the article/Supplementary material.

## Ethics statement

The animal study was approved by the animal welfare and ethical committee of Hainan University, Hainan, China (HNUAUCC-2021-00083). All procedure performed in study involving animals were in accordance with the ethical standards of the institution or practice at which the studies were conducted. The study was conducted in accordance with the local legislation and institutional requirements.

## Author contributions

LF: Writing – original draft, Visualization, Funding acquisition, Formal analysis, Data curation. JP: Writing – review & editing, Resources, Investigation, Data curation. JZ: Writing – review & editing, Supervision, Funding acquisition, Conceptualization. GG: Writing – review & editing, Methodology, Funding acquisition. NY: Writing – review & editing, Methodology, Funding acquisition. XL: Writing – review & editing, Methodology, Data curation. MN: Writing – review & editing. JZh: Writing – review & editing, Project administration, Funding acquisition, Conceptualization.
