# Supplementary material for: An outbreak of Providencia rettgeri bacteremia at a Ptyas mucosus farm in Hainan, China
Source: Front Microbiol. 2024 Jul 11;15:1353603. doi: 10.3389/fmicb.2024.1353603 (PMC11269246; doi:10.3389/fmicb.2024.1353603)
Supplement: Supplementary file 1 [file Table_1.docx]

Supplementary Information

**Table S1 Primers used for the detection of antimicrobial-resistance genes**

| **Resistance gene** | **Primer** | **sequence (5´- 3´)** | **Size bp** | **Reference** |
| --- | --- | --- | --- | --- |
| *bla*_TEM_ | *bla*_TEM_-F | GAGTATTCAACATTTTCGT | 857 | (Lin et al., 2016) |
|  | *bla*_TEM_-R | ACCAATGCTTAATCAGTGA |  |  |
| *bla*_SHV_ | *bla*_SHV_-F | TCGCCTGTGTATTATCTCCC | 768 | (Lin et al., 2016) |
|  | *bla*_SHV_-R | CGCAGATAAATCACCACAATG |  |  |
| *bla*_CTXM_ | *bla*_CTXM_-F | AATCACTGCGTCAGTTCAC | 701 | (Maynard et al., 2004) |
|  | *bla*_CTXM_-R | TTTATCCCCCACAACCCAG |  |  |
| *bla*_OXA_ | *bla*_OXA_-F | GCAGCGCCAGTGCATCAAC | 198 | (Lin et al., 2016) |
|  | *bla*_OXA_-R | CCGCATCAAATGCCATAAGTG |  |  |
| *Tet*(A) | *Tet*(A)-F | GCTACATCCTGCTTGCCTTC | 210 | (Lin et al., 2016) |
|  | *tet*(A)-R | CATAGATCGCCGTGAAGAGG |  |  |
| *tet*(B) | *tet*(B)-F | TTGGTTAGGGGCAAGTTTTG | 659 | (Lin et al., 2016) |
|  | *tet*(B)-R | GTAATGGGCCAATAACACCG |  |  |
| *tet*(E) | *tet*(E)-F | ATGAACCGCACTGTGATGATG | 744 | (Lin et al., 2016) |
|  | *tet*(E)-R | ACCGACCATTACGCCATCC |  |  |
| *aph*AI-IAB | *aph*AI-IAB-F | ATGGGCTCGCGATAATGTC | 600 | (Lin et al., 2016) |
|  | *aph*AI-IAB-R | CTCACCGAGGCAGTTCCAT |  |  |
| *aac*(3)-IIa | *aac*(3)-IIa -F | CGGAAGGCAATAACGGAG | 740 | (Pathirana et al., 2018; Lin et al., 2016) |
|  | *aac*(3)-IIa -R | TCGAACAGGTAGCACTGAG |  |  |
| *aac*(6’)-lb | *aac*(6’)-lb-F | TTGCGATGCTCTATGAGTGGCTA | 482 | (Pathirana et al., 2018; Lin et al., 2016) |
|  | *aac*(6’)-lb-R | CTCGAATGCCTGGCGTGTTT |  |  |
| *str*A-B | *str-A* | TATCTGCGATTGGACCCTCTG | 538 | (Pathirana et al., 2018; Lin et al., 2016) |
|  | *str-B* | CATTGCTCATCATTTGATCGGCT |  |  |
| *arm*A | *arm*A-F | AGGTTGTTTCCATTTCTGAG | 591 | (Pathirana et al., 2018; Lin et al., 2016) |
|  | *arm*A-R | TCTCTTCCATTCCCTTCTCC |  |  |

**Table S2 Primers used for virulence genes detection**

| **virulence name** | **Primer** | **sequence (5´- 3´)** | **Size bp** | **Reference** |
| --- | --- | --- | --- | --- |
| *mrp*A | *mrp*A-F | TTCTTACTGATAAGACATTG | 565 | (Abbas et al., 2015) |
|  | *mrp*A-R | ATTTCAGGAAACAAAAGATG |  |  |
| *ure*C | *ure*C-F | CCGGAACAGAAGTTGTCGCTGGA | 533 | (Abbas et al., 2015) |
|  | *ure*C-F | GGGCTCTCCTACCGACTTGATC |  |  |
| *hpm*A | *hpm*A-F | GTTGAGGGGCGTTATCAAGAGTC | 709 | (Cestari et al., 2013) |
|  | *hpm*A-R | GATAACTGTTTTGCCCTTTTGTGC |  |  |
| *hly*A | *hly*A-F | AACAAGGATAAGCACTGTTCTGGCT | 1177 | (Cestari et al., 2013) |
|  | *hly*A-R | ACCATATAAGCGGTCATTCCCGTCA |  |  |
| *zap*A | *zap*A-F | ACCGCAGGAAAACATATAGCCC | 540 | (Stankowska et al., 2008) |
|  | *zap*A-R | GCGACTATCTTCCGCATAATCA |  |  |
| *rsb*A | *rsb*A-F | TTGAAGGACGCGATCAGACC | 467 | (Abbas et al., 2015) |
|  | *rsb*A-R | ACTCTGCTGTCCTGTGGGTA |  |  |
| *lux*S | *lux*S-F | GTATGTCTGCACCTGCGGTA | 464 | (Abbas et al., 2015) |
|  | *lux*S-R | TTTGAGTTTGTCTTCTGGTAG TGC |  |  |

**Table S3 Biochemical characteristics of the isolates**

| **Characteristic** | **Isolates (100%)** |
| --- | --- |
| O-nitrobenzene-galactoside | - |
| Arginine dihydrolase | - |
| Lysine | - |
| Ornithine decarboxylase | - |
| Sodium citrate | + |
| Sodium thiosulfate | - |
| Urea | + |
| Tryptophan | + |
| Indole | + |
| Pyruvate | - |
| Gelatinase | - |
| Glucose | + |
| Mannitol | + |
| Inositol | + |
| Sirbitol | - |
| Rhamnose | + |
| Sucrose | - |
| Melibiose | - |
| Amygdalin | + |
| Arabinose | - |
| Oxidase | - |

**References**

1. Pathirana, H., Shin, G.W., Wimalasena, S., Hossain, S., De Silva, B.C.J., Dahanayake, P.S., et al.(2018). Incidence of antibiogram, antibiotic resistance genes and class 1 and 2 integrons in tribe Proteeae with IMP27 gene for the first time in Providencia sp. isolated from pet turtles. *Lett Appl Microbiol*. 67, 620-27. doi:10.1111/lam.13077.

2. Abbas, K.F., Khafaji, J.K.A., and Al-Shukri, M.S. (2015). Molecular Detection of Some Virulence Genes in Proteus Mirabilis Isolated from Hillaprovince. *IJRSB*. 3,85-89. doi:10.11648/j. avs. 20150301.15.

3. Lin, M., Wu, X., Yan, Q., Ma, Y., Huang, L., Qin, Y., et al.(2016). Incidence of antimicrobial-resistance genes and integrons in antibiotic-resistant bacteria isolated from eels and aquaculture ponds. *Dis Aquat Org*. 120, 115-23. doi:10.3354/dao03013.

4. Maynard, C., Bekal, S., Sanschagrin, F., Levesque, R.C., Brousseau, R., Masson, L.,et al. (2004). Heterogeneity among virulence and antimicrobial resistance gene profiles of extraintestinal Escherichia coli isolates of animal and human origin. *J Clin Microbiol*. 42, 5444-52. doi:10. 1128/JCM. 42. 12. 5444-5452.2004.

5. Cestari, S.E., Ludovico, M.S., Martins, F.H., da Rocha, S.P., Elias, W.P., and Pelayo, J.S. (2013). Molecular detection of HpmA and HlyA hemolysin of uropathogenic Proteus mirabilis. *Curr Microbiol*. 67, 703-7. doi:10.1007/s00284-013-0423-5.

6. Stankowska, D., Kwinkowski, M., and Kaca, W. (2008). Quantification of Proteus mirabilis virulence factors and modulation by acylated homoserine lactones. *J Microbiol Immunol Infect*. 41, 243-53.
